# Class Effect Unveiled: PPARγ Agonists and MEK Inhibitors in Cancer Cell Differentiation

**DOI:** 10.3390/cells13171506

**Published:** 2024-09-09

**Authors:** Rakefet Ben-Yishay, Opher Globus, Nora Balint-Lahat, Sheli Arbili-Yarhi, Neta Bar-Hai, Vered Bar, Sara Aharon, Anna Kosenko, Adi Zundelevich, Raanan Berger, Dana Ishay-Ronen

**Affiliations:** 1 Oncology Institute, Sheba Medical Center, Ramat Gan 5262000, Israel; rakefetruth.benyishay@sheba.health.gov.il (R.B.-Y.); opher.globus@sheba.health.gov.il (O.G.); sheli.arbiliyarhi@sheba.health.gov.il (S.A.-Y.); neta.gochman@sheba.health.gov.il (N.B.-H.); 2 Institute of Pathology, Sheba Medical Center, Ramat Gan 5262000, Israel; nora.balintlahat@sheba.health.gov.il; 3 Faculty of Medicine, Tel Aviv University, Tel Aviv 6997801, Israel; 4 Curesponse Ltd., Rehovot 7670102, Israel; vered@curesponse.tech (V.B.); anna@curesponse.tech (A.K.);

**Keywords:** breast cancer, differentiation therapy, PPARγ agonist, thiazolidinediones, MEK inhibitor, EMT, adipogenesis

## Abstract

Epithelial-to-mesenchymal transition (EMT) plays a major role in breast cancer progression and the development of drug resistance. We have previously demonstrated a trans-differentiation therapeutic approach targeting invasive dedifferentiated cancer cells. Using a combination of PPARγ agonists and MEK inhibitors, we forced the differentiation of disseminating breast cancer cells into post-mitotic adipocytes. Utilizing murine breast cancer cells, we demonstrated a broad class effect of PPARγ agonists and MEK inhibitors in inducing cancer cell trans-differentiation into adipocytes. Both Rosiglitazone and Pioglitazone effectively induced adipogenesis in cancer cells, marked by PPARγ and C/EBPα upregulation, cytoskeleton rearrangement, and lipid droplet accumulation. All tested MEK inhibitors promoted adipogenesis in the presence of TGFβ, with Cobimetinib showing the most prominent effects. A metastasis ex vivo culture from a patient diagnosed with triple-negative breast cancer demonstrated a synergistic upregulation of PPARγ with the combination of Pioglitazone and Cobimetinib. Our results highlight the potential for new therapeutic strategies targeting cancer cell plasticity and the dedifferentiation phenotype in aggressive breast cancer subtypes. Combining differentiation treatments with standard therapeutic approaches may offer a strategy to overcome drug resistance.

## 1. Introduction

Cancer cells have the remarkable ability to change their phenotype and adapt to external signals from their microenvironment, a phenomenon known as cancer cell plasticity [1,2,3]. Epithelial-to-mesenchymal transition (EMT) and its reversal mesenchymal-to-epithelial transition (MET) are essential processes in embryonic development and can be reactivated and utilized by cancer cells to enhance cellular plasticity and malignant progression [1,4]. EMT in breast cancer is particularly associated with tumor invasion, metastasis and drug resistance [3,5].

Our previous work demonstrated a novel approach to overcoming cancer cell plasticity by converting invasive breast cancer cells into post-mitotic functional adipocytes through the combined use of a PPARγ agonist (Rosiglitazone) and a MEK inhibitor (Trametinib). The trans-differentiation approach effectively repressed breast cancer progression and metastasis formation in murine and human models [6].

EMT is regulated by several signaling pathways, with TGFβ playing a central role. TGFβ induces EMT in breast cancer cells, resulting in a dedifferentiated cell state with increased plasticity [7,8]. This process involves the upregulation of EMT transcription factors such as Zeb1, Zeb2, and Klf4, which also regulate adipogenesis [7,9,10,11]. However, TGFβ also activates the non-canonical MEK-ERK pathway, which promotes EMT, cell proliferation, and survival, thereby impeding differentiation processes [12,13]. ERK was shown to phosphorylate PPARγ, thereby impeding its activity and adipogenesis regulation [14,15]. We previously demonstrated that inhibiting the MEK-ERK pathway with a MEK inhibitor, in combination with a PPARγ agonist, facilitates the differentiation of breast cancer cells into adipocytes [6,9].

Adipogenesis is the process by which preadipocytes differentiate into mature adipocytes. This process is regulated by several transcription factors, including CCAAT–enhancer binding protein α (C/EBPα) and PPARγ, which are considered master regulators of adipogenesis [16,17]. PPARγ activation is essential for adipogenesis, as it initiates the transcriptional cascade that leads to the terminal differentiation of adipocytes. C/EBPα also plays a crucial role, although it is not sufficient to induce adipogenesis in the absence of PPARγ [16].

TGFβ plays a significant role in inhibiting adipogenesis by interacting with key adipogenic pathways. It activates canonical SMAD3, which binds to C/EBPs and inhibits their transcriptional activity, thereby preventing the upregulation of PPARγ and the adipogenic program [13]. TGFβ-mediated inhibition of non-cancer adipogenesis is primarily linked to its canonical signaling pathway through SMAD proteins, particularly SMAD3 [13]. Moreover, TGFβ activation of the non-canonical MEK-ERK pathway further interferes with adipogenesis by preventing the differentiation of preadipocytes into mature adipocytes [6,15].

Pioglitazone and Rosiglitazone are thiazolidinediones (TZDs), clinically available synthetic PPARγ agonists with potent insulin-sensitizing effects, and have been extensively used in the treatment of type 2 diabetes [18]. MEK inhibitors, such as Cobimetinib, Binimetinib, Selumetinib, and Trametinib, are targeted therapies that inhibit the MEK-ERK signaling pathway, which is often dysregulated in-cancer [19,20,21,22].

Our current study aims to investigate whether the induction of adipogenesis by the combination of PPARγ agonists and MEK inhibitors is a class effect, rather than being specific to Trametinib and Rosiglitazone. We treated murine breast cancer cells with Pioglitazone alone and in combination with various clinically available MEK inhibitors, including Cobimetinib, Binimetinib, Selumetinib, and Trametinib, at different dose levels to evaluate their effectiveness in inducing adipogenesis. Notably, Selumetinib is a MEK inhibitor that is clinically available as a pediatric drug for neurofibromatosis type 1, while the rest of the tested MEK inhibitors are routinely used in medical oncology. We further demonstrate the possibility of inducing adipogenesis and PPARγ activation in patient-derived ex vivo tissue cultures.

Demonstrating a class effect of PPARγ agonists with MEK inhibitors in promoting adipogenesis is significant, as it suggests a broader therapeutic potential for this combination in cancer treatment. The ability to generalize this effect across multiple MEK inhibitors would indicate that the mechanism of action is not limited to specific drug pairs, but rather is a fundamental biological interaction between PPARγ activation and MEK inhibition. This could expand the range of available therapeutic options and provide insights into the underlying mechanisms of cancer cell plasticity and differentiation, offering new targets for drug development. By leveraging the synergistic effects of these drugs, we can develop new strategies to inhibit cancer cell proliferation and promote differentiation into less malignant cell types with the potential to improve clinical outcomes for patients with aggressive cancers. 

## 2. Materials and Methods

### 2.1. Cell Culture

MTΔECad (a kind gift from Christofori lab, University of Basel) were previously described [23]. Specifically, MTflECad cells isolated from a lymph node metastasis of a MMTV-Neu;Cdh1fl/fl tumor-bearing mouse [24] were infected with an adenovirus-expressing Cre recombinase, obtaining the E-cadherin-deficient cell line MTΔECad [25]. 

Cells were cultured in a high-glucose Dulbecco’s modified Eagle medium (DMEM (Sartorius, Göttingen, Germany; 01-055-1A)) supplemented with 10% Fetal Bovine Serum (FBS; A5256701; ThermoFisher, Waltham, MA, USA), ×1 Penicillin/streptomycin (L0022-100; Biowest, Nuaillé, France), and 2 mM L-Glutamine (X0550-100; Biowest, Nuaillé, France). Cells were grown at 37 °C, 5% CO_2_, and 95% humidity.

### 2.2. Cancer Trans-Differentiation into Adipocytes

For standard cancer adipogenesis experiments, cells were seeded at a density of 20,000 cells/cm^2^ in an 18-well μ Slide (81816; ibidi, Gräfelfing, Germany) and incubated overnight at 37 °C in 5% CO_2_. Then, cells were treated with 200 ng/mL human recombinant BMP2 (B3555, Sigma, Saint Louis, MO, USA) for 3 days, with 200 ng/mL BMP2 and 2 μM Rosiglitazone (AG-CR1-3570-M010; Adipogen, San Diego, MA, USA) or Pioglitazone (71745-50; Cayman Chemical, Ann Arbor, MI, USA) as indicated for 4 days, and with a medium containing 2 μM Rosiglitazone or Pioglitazone as indicated for an additional 3 days.

For MEK inhibitor experiments, cells were seeded in an ibidi 18-well μ Slide at a density of 30,000 cells/cm^2^ and incubated overnight. Then, cells were treated for 3 days with 200 ng/mL human recombinant BMP2, 2 ng/mL TGFβ (R&D Systems, Minneapolis, MN, USA; 240-B), and MEK inhibitors (Trametinib; BNT-8123, Selumetinib; BNS-4490, Binimetinib; B-2332, Cobimetinib; BNC-1100; Lc Laboratories, Woburn, MA, USA) as indicated. Then, cells were treated for 4 days with BMP2, TGFβ, MEK inhibitors, and 2 μM Rosiglitazone or 20 μM Pioglitazone. Finally, cells were treated for 3 days with Rosiglitazone or Pioglitazone and fixed with 4% Paraformaldehyde (PFA). Control cells were treated with a medium containing DMSO.

### 2.3. Cell Proliferation Assay (EdU)

MTΔECad cells were treated for 10 days with 200 ng/mL BMP2 and 2 μM Rosiglitazone (Rosi) or 10 μM Pioglitazone (Pio), with or without 2 ng/mL TGFβ and MEK inhibitors (0.5 ng/mL Trametinib, 400 μM Binimetinib, 0.25 μM Selumetinib, or 0.25 μM Cobimetinib). On day 7 of the adipogenesis protocol, cells were incubated for 72 h with 5 μM Click-iT EdU (C10337, ThermoFisher, Waltham, MA, USA). The ClickIT reaction was performed according to the manufacturer’s protocol, and nuclear staining was performed with 4′,6-diamidino-2-phenylindole (DAPI; D1306; Invitrogen, Waltham, MA, USA) for 10 min. Cells were washed with PBS, mounted with ibidi mounting medium (IBD-50001; ibidi, Gräfelfing, Germany), and imaged with a fluorescent microscope. 

### 2.4. Ex Vivo Cultures of Tumor Tissues

Tumor tissue was obtained from a metastatic lymph node core needle biopsy of a 37-year-old metastatic triple-negative breast cancer patient after her informed consent had been obtained (Ethic approval no. 7188-20-smc, date of approval: 10 May 2020). The tissue was cut into 250 μm slices using a vibratome (VF300, Precisionary Instruments, Ashland, MA, USA). 

A sample of the tissue was fixed immediately in 4% paraformaldehyde (PFA) as a reference and analyzed for viability within 24 h. The rest of the slices were placed in 12- or 24-well plates on titanium grids with 4 mL of DMEM/F12 medium (D6421, Sigma, Saint Louis, MO, USA) [supplemented with 5% fetal calf serum (FCS) (10270-106, Gibco, Waltham, MA, USA), penicillin 100 IU/mL with streptomycin 100 μg/mL (15140-122, ThermoFisher, Waltham, MA, USA), Amphotericin B (A2942, Sigma, Saint Louis, MO, USA) 2.5 μg/mL, 50 mg/mL Gentamicin sulfate (G1397, Sigma), and L-glutamine 100 μL/mL (03-020-1B, Sartorius, Göttingen, Germany)]. The tissue slices were then cultured at 70 rpm on an orbital shaker (TOU-120) at 37 °C, 5% CO_2_, and 80% O_2_. One day after sectioning, tumor sections were treated with drugs and cultured for 96 h with a medium change after 48 h. At the end of the incubation period, the tissue sections were fixed overnight with 4% PFA followed by a formalin-fixed paraffin embedding (FFPE). Drug concentrations: Cobimetinb 1.25–2.5 μM; Pioglizonate (20–100 μM). 

### 2.5. Immunohistochemistry (IHC)

IHC staining was performed on 4 μm FFPE sections using the Leica Bond max system (Leica Biosystems Newcastle Ltd., Nussloch, Germany). Slides were baked for 30 min at 60° C, dewaxed, and pretreated for 20 min with epitope-retrieval solution (ER2, Leica Biosystems Newcastle Ltd., Nussloch, Germany), followed by incubation with an anti-PPARγ antibody (Santa Crus SC-7273, 1:50). Detection was performed using the Leica Bond Polymer Refine HRP kit (Leica Biosystems Newcastle Ltd., Nussloch, Germany). All slides were counter-stained with Hematoxylin. 

### 2.6. Immunofluorescence

Cells were plated in an 18-well μ Slide (81816; ibidi, Gräfelfing, Germany) and covered with an appropriate growth medium overnight. Following treatments, cells were fixed in 4% paraformaldehyde for 20 min, permeabilized with 0.5% Triton X100 (93443, Sigma Aldrich, Saint Louis, MO, USA) in phosphate-buffered saline (PBS; 02-023-1A; Sartorius, Göttingen, Germany) for 2 min, and blocked with 5% Bovine serum albumin (BSA; Avantor; 0332-50G) in PBS for 20 min. The cells were incubated with a primary antibody diluted 1:100 in PBS (Rabbit anti PPARγ, CST-2443S; Rabbit anti C/EBPα, CST-8178S; or rabbit anti Perilipin, CST-9349S; Cell Signaling, Danvers, MA, USA) for 1 h at room temperature (RT), and washed three times for 5 min with PBS at RT. Then, cells were incubated with a secondary antibody diluted 1:500 in PBS (Alexa Fluor 647 labeled gout anti Rabbit (ab150083; abcam, Cambridge Biomedical Campus, Cambridge, UK); Alexa Fluor 488 labeled Goat Anti-Rabbit (ab150077; abcam), or Alexa Fluor 555 labeled Donkey Anti-Rabbit (ab150062; abcam)) for 1 h at RT, together with Phalloidin-iFluor 647 Reagent (ab176759; abcam) (1:1000) or BODIPY FL C12 (D3822; Thermo Fisher, Waltham, MA, USA) (1:5000) as indicated in the figure legends. The cells were washed three times for 5 min with PBS at RT, and stained with 4′,6-diamidino-2-phenylindole (DAPI; D1306; Invitrogen, Waltham, MA, USA) for 10 min to mark the cell nuclei. Finally, the cells were rapidly washed with PBS and covered with ibidi mounting medium (IBD-50001; ibidi, Gräfelfing, Germany) or PBS.

### 2.7. Microscopy

Confocal and widefield fluorescent imaging was performed with a TCS SP8 Leica Confocal microscope, equipped with a Leica DFC9000 GT camera (Leica Microsystems, Nussloch, Germany). 

IHC slides were scanned at ×40 magnification using VENTANA^®^ DP 200 slide scanner (Roche Diagnostics, Basel, Switzerland).

### 2.8. Image Processing and Analysis

Image processing (i.e., Crope, pseudo-coloring, merge channels) was performed with LAS X software (3.7.2.22383) and the Fiji processing software package of ImageJ (1.51n). For quantification of PPARγ- and C/EBPα-positive cells, the DAPI and PPARγ/C/EBPα channels of individual fields were thresholded by ImageJ, and single nuclei were counted using the ‘Analyze Particles’ feature. The percentage of PPARγ/C/EBPα count out of the total DAPI count was calculated for every field, and the average percentages for every condition were plotted using Microsoft Excel 2016 software. Analysis of PPARy-positive cells in IHC images was performed using QuPath 5.0.

### 2.9. Statistical Analysis

Statistical comparisons between groups were performed using a two-tailed *t* test in Microsoft Excel software.

## 3. Results

### 3.1. Pioglitazone Can Effectively Substitute Rosiglitazone and Induce the Trans-Differentiation of Cancer Cells into Adipocytes

The previously published epithelial MTflECad (with floxed E-cadherin) cells are derived from a murine MMTV-neu-driven primary tumor. Upon Cre recombination, the cells undergo E-cadherin ablation and become irreversibly mesenchymal, generating the MTΔECad cells [6,23]. MTΔECad cells can undergo adipogenesis by activating MET with BMP2 (BMP2 is required only in in vitro protocols [6]) and Rosiglitazone. When TGFβ is added to the protocol, a combination with a MEK inhibitor is required to induce adipogenesis [6]. Thus, these cells serve as an efficient model for studying isolated drug effects. 

**Figure 1:**
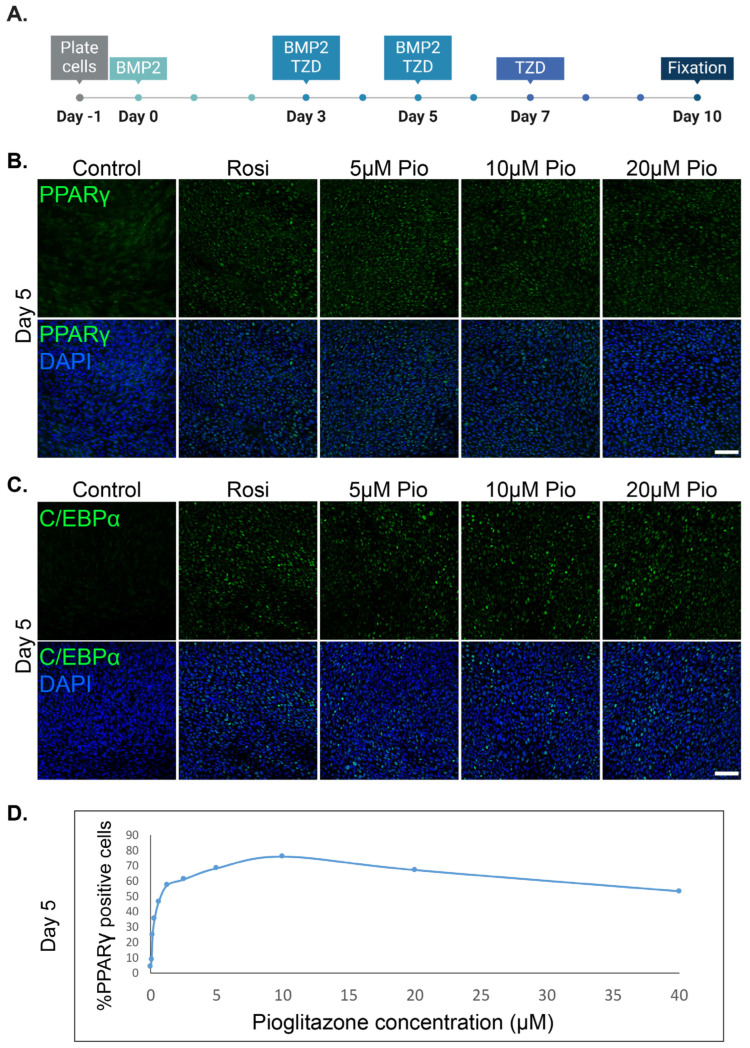
Pioglitazone upregulates adipogenesis-related transcription factors in murine breast cancer cells in a dose-dependent manner. (**A**) A scheme describing the optimized protocol to induce MTΔECad cells’ trans-differentiation into adipocytes with BMP2 and thiazolidinediones (TZDs). (**B**,**C**). MTΔECad cells were treated to induce adipogenesis as described in (**A**) for 5 days with BMP2 and either 2 μM Rosiglitazone (Rosi) or 5, 10, or 20μM Pioglitazone (Pio) as indicated. Control cells were treated with DMSO. Cells were then fixed and immunostained with antibodies against the adipogenesis-driving transcription factors PPARy (**B**) and C/EBPα (**C**) (top, green) and imaged with a confocal microscope. Merged images with DAPI nuclear counterstaining (blue) are presented in the bottom rows. Bars = 50 µm. (**D**) A graph demonstrating the percentage of PPARy-positive cells out of MTΔECad cells treated for adipogenesis with BMP2 and increasing concentrations of Pioglitazone for 5 days.

To determine a generalized class effect enabling adipogenesis, we utilized established protocols to induce the trans-differentiation of cancer cells into adipocytes. Murine MTΔECad breast cancer cells were induced to undergo trans-differentiation into adipocytes, as previously described [6]. The established in vitro protocol combines BMP2 with Rosiglitazone, a synthetic PPARγ agonist from the TZD family. To study the broad effect of TZDs on cancer’s trans-differentiation into adipocytes, we tested the effect of gradually increasing the dose of Pioglitazone on adipogenesis induction (Figure 1A). The two major adipogenesis transcription factors, PPARγ and C/EBPα, were upregulated as demonstrated by immunolabeling and confocal microscopy visualization (Figure 1). The effect of Pioglitazone on PPARγ upregulation was measured after 5 days of adipogenesis induction and revealed an ideal dose range from 5 to 20 μM (Figure 1D). 

**Figure 2:**
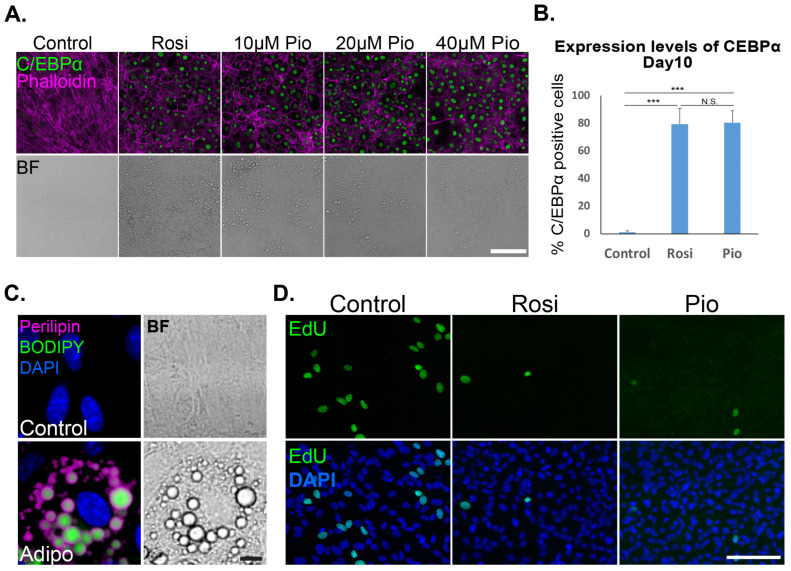
Pioglitazone induces trans-differentiation of murine breast cancer cells into well-differentiated adipocytes. (**A**) Adipogenesis was induced in MTΔECad cells for 10 days with 200 ng/mL BMP2 and either 2 μM Rosiglitazone (Rosi) or 10, 20, or 40 μM Pioglitazone (Pio) as indicated. Control cells were treated with DMSO. Cells were immunostained with anti C/EBPα antibody (green) and counterstained with Phalloidin to label F-actin (Magenta), visualizing actin rearrangement from stress fibers into cortical structures. Brightfield (BF) images demonstrate the formation of lipid droplets in the cytoplasm of treated cells. Bar = 100 µm. (**B**) Quantification of the percentage of C/EBPα-expressing cells in DMSO (control), BMP2 + Rosiglitazone (Rosi), or BMP2 + Pioglitazone (Pio) treated cells. After 10 days of cancer adipogenesis treatment, cells were fixed, immunostained with an anti C/EBPα antibody and DAPI to mark cell nuclei, and visualized with confocal microscopy. C/EBPα-positive cells were quantified *n* = 3. A two tailed t-test indicated no statistically significant difference between Rosi and Pio treatments (***, *p* < 0.001; N.S., *p* > 0.05). (**C**) MTΔECad Cells treated for 10 days with DMSO (control) or with BMP2 + Pioglitazone (Adipo) were immunostained with an antibody against the specific adipocyte marker Perilipin (a lipid droplet membrane protein, Magenta), and counterstained with BODIPY to visualize lipid droplets (green) and with DAPI (blue). Brightfield (BF) images of the same cells are shown on the right. Bar = 10 μm. (**D**) Representative images of MTΔECad cells treated for 10 days with DMSO (control), BMP2 + Rosiglitazone (Rosi), and BMP2 + Pioglitazone (Pio). On day 7 of adipogenesis treatment, cells were incubated with 5-ethynyl-20-deoxyuridine (EdU) for 72 hours to label proliferating cells (green). DAPI stain is shown in blue. Bar = 100 µm.

Next, we asked whether the efficient adipogenesis transcription factors upregulation induced with Pioglitazone results in mature adipocyte trans-differentiation in a similar manner to Rosiglitazone [6]. Indeed, the upregulated C/EBPα correlated with a phenotypic remodeling of the cytoskeleton, shifting from mesenchymal stress fibers into cortical actin reorganization (Figure 2A,B). A specific mature adipocyte marker detected during cancer adipogenesis is the accumulation of lipid droplets organized with the lipid droplet membrane protein Perilipin (Figure 2C). Furthermore, during cancer cells’ trans-differentiation, cancer cells undergo growth arrest typical for mature adipocytes (Figure 2D).

### 3.2. Class Effect of MEK Inhibitors Facilitates Cancer Trans-Differentiation in the Presence of TGFβ

TGFβ is a potent inducer of EMT in vitro and in vivo. Yet, TGFβ impairs the development of adipose tissue and inhibits adipogenesis [13]. We previously demonstrated the efficient inhibition of ERK phosphorylation with PD98059 and an FDA-approved MEK inhibitor, Trametinib. Here, we compared the effect of clinically available MEK inhibitors with different toxicity profiles on cancer cells-induced adipogenesis. 

MTΔECad cells undergo irreversible EMT upon Cre recombination as mentioned, and do not require TGFβ to induce cell dedifferentiation. Thus, MTΔECad cells can be utilized to specifically investigate the inhibitory effect of TGFβ on cancer cells’ trans-differentiation into adipocytes, regardless of its role in EMT induction [6]).

**Figure 3:**
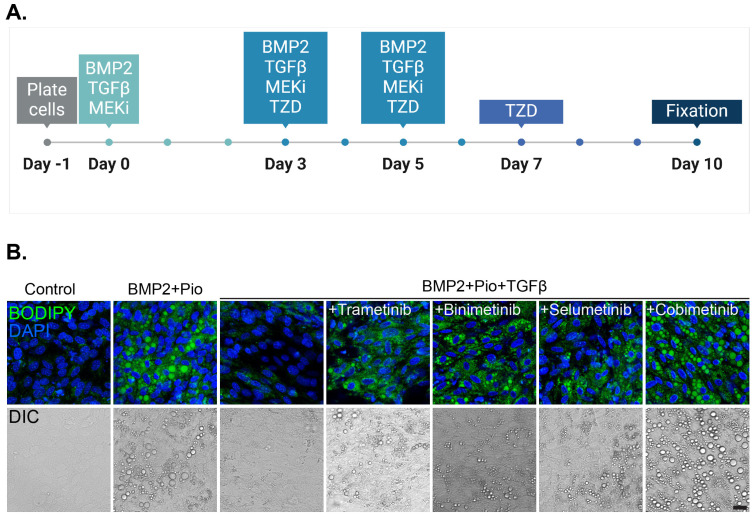
Class effect of MEK inhibitors enables cancer trans-differentiation in the presence of TGFβ (**A**) Schematic description of induction protocol for MTΔECad cells’ cancer adipogenesis in the presence of TGFβ with BMP2, thiazolidinediones (TZDs), and an MEK inhibitor (MEKi). (**B**) MTΔECad cells were treated as indicated in (**A**) with BMP2 + Pioglitazone (BMP2 + Pio), BMP2 + Pioglitazone and TGFβ, or BMP2 + Pioglitazone + TGFβ together with an MEK inhibitor (0.5 ng/mL Trametinib, 400 nM Binimetinib, 0.25 μM Selumetinib, or 0.25 μM Cobimetinib) as indicated. Control cells were treated with DMSO. After 10 days, cells were fixed, stained with BODIPY to mark lipid droplets (green) and with DAPI (blue), and imaged with a confocal microscope. DIC images of the same fields are shown at the bottom. Bar = 20 μm.

MTΔECad cells were induced to undergo adipogenesis in comparison to adipogenesis inhibition by TGFβ. As indicated, several FDA-approved MEK inhibitors were tested for their effect on adipogenesis induction, including Selumetinib, a drug used for neurofibromatosis in pediatric patients. Adipogenesis induction was assessed in increasing doses of MEK inhibitors (Figure 3 and Figure S1). While all tested MEK inhibitors were able to induce adipogenesis, we chose to focus on Cobimetinib for further evaluations due to the accumulation of prominent lipid droplets and proliferation inhibition (Figure 3, Figures S1 and S2).

**Figure 4:**
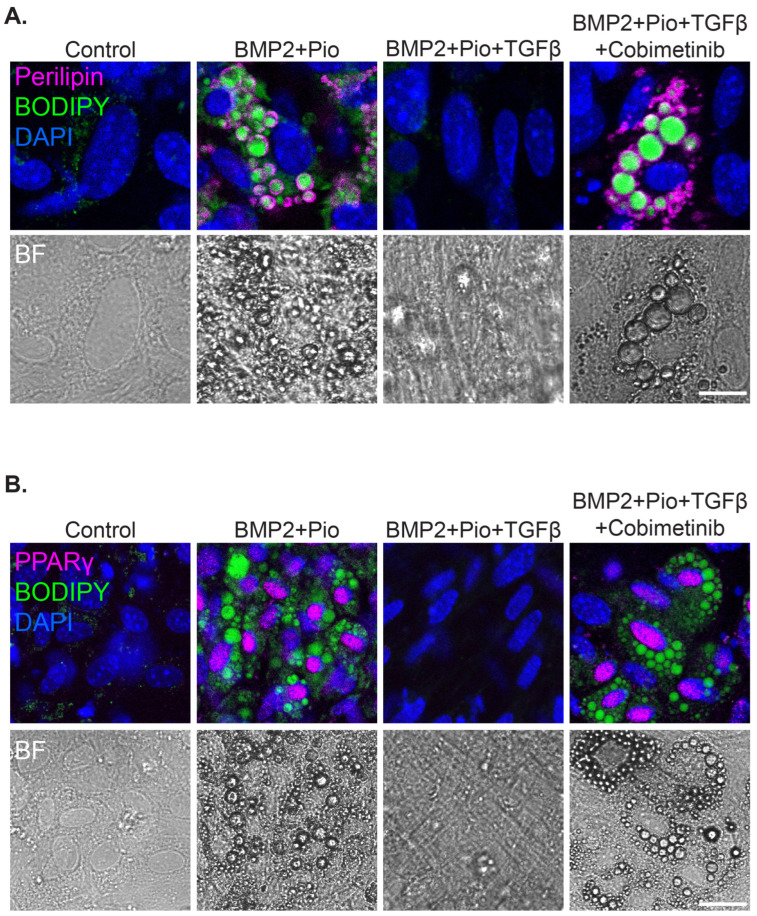
Pioglitazone and Cobimetinib can induce trans-differentiation of murine breast cancer cells into mature adipocytes in the presence of TGFβ. MTΔECad cells were treated for 10 days with BMP2 + Pioglitazone (BMP2 + Pio), BMP2 + Pioglitazone and TGFβ, or BMP2 + Pioglitazone + TGFβ together with the MEK inhibitor Cobimetinib. Cells were immunostained with antibodies against Perilipin (**A**) or PPARγ (**B**) (Magenta) and counterstained with BODIPY to mark lipid droplets (green) and DAPI (blue). Brightfield images of the same fields are shown in the bottom (BF). Bars = 20 µm.

Cobimetinib induced PPARγ upregulation and lipid droplet accumulation with Perilipin expression when combined both with Rosiglitazone as well as with Pioglitazone (Figure 4 and Figure S3).

**Figure 5:**
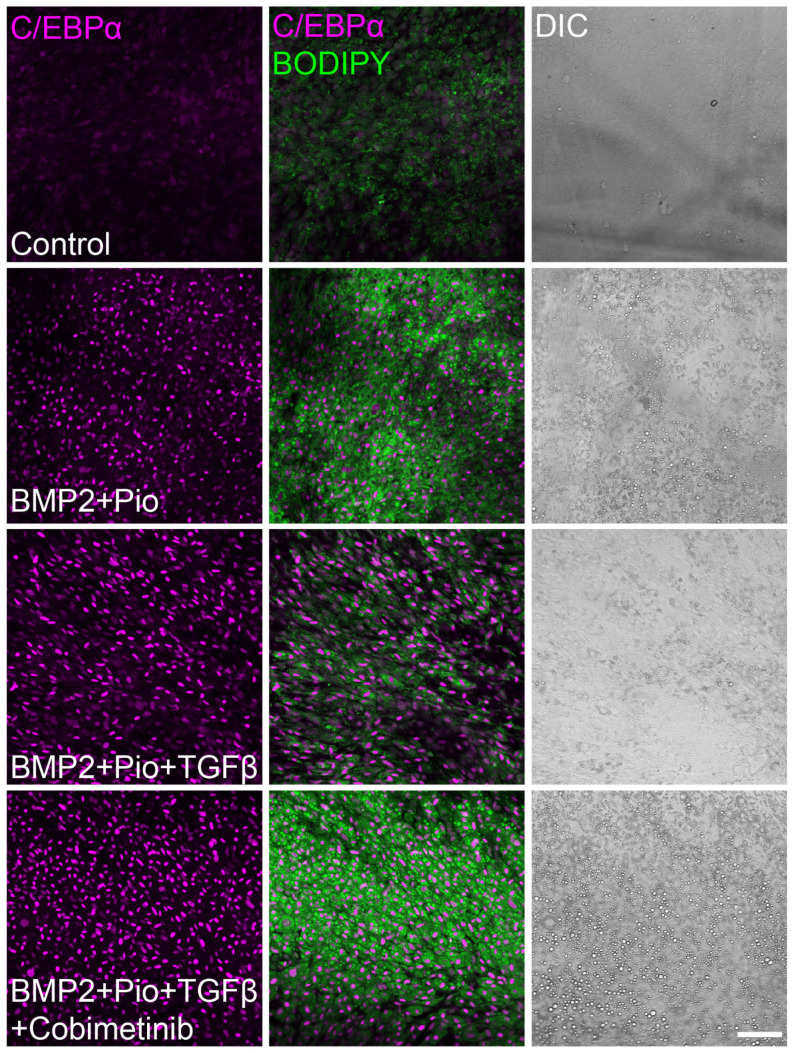
C/EBPα is upregulated in cancer cells-induced adipogenesis regardless of TGFβ. Confocal imaging of MTΔECad cells treated for 10 days with BMP2 + Pioglitazone (BMP2 + Pio), BMP2 + Pioglitazone and TGFβ, or BMP2 + Pioglitazone + TGFβ together with Cobimetinib. Control cells were treated with DMSO. Cells were immunostained with anti-C/EBPα antibody (Magenta) and counterstained with BODIPY for lipid droplets labeling (green). DIC images are shown on the right. Bar = 100 µm.

Intriguingly, TGFβ impedes adipogenesis induction in cancer cells by inhibiting PPARγ expression but not C/EBPα (Figure 5), thus suggesting a direct effect of TGFβ-induced MEK-ERK activation on PPARγ regulation. Yet, these results highlight the essential role of PPARγ activation in adipogenesis induction even in the presence of C/EBPα.

### 3.3. A Synergistic Effect of Pioglitazone and Cobimetinib in PPARγ Upregulation in Patient-Derived Ex Vivo Tumor Culture

**Figure 6:**
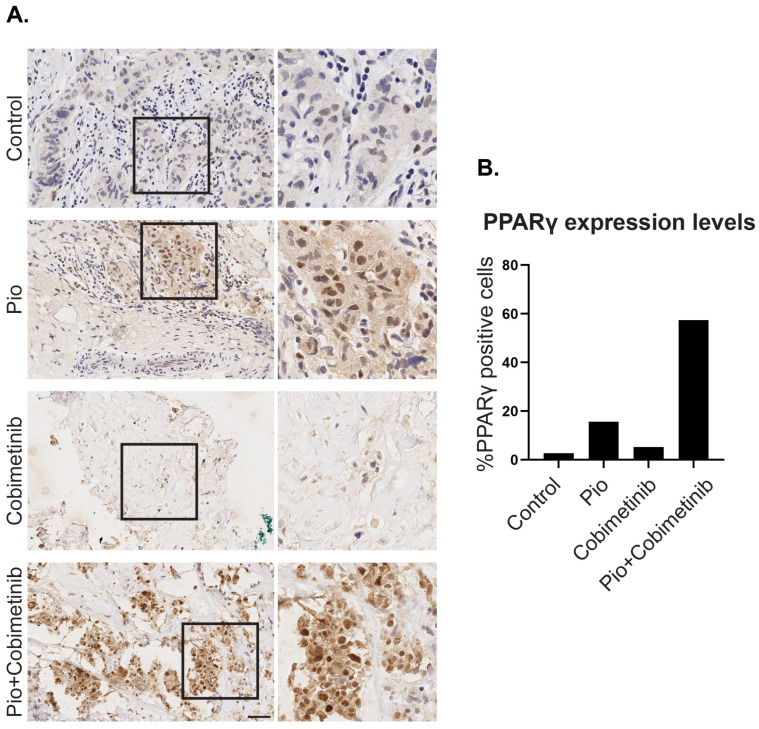
Upregulation of PPARγ in ex vivo patient-derived breast cancer culture. (**A**) Three-dimensional tissues obtained from a core needle biopsy from the tumor of a triple-negative breast cancer patient were treated for 5 days with DMSO (control), Pioglitazone (Pio), Cobimetinib, or with a combination of Pioglitazone and Cobimetinib (Pio + Cobimetinib). Tissues were fixed and IHC staining with an antibody against PPARγ was performed (brown). Counterstaining with Hematoxylin is depicted in blue. Enlargement of the squared areas is shown on the right. Bar = 50 μm. (**B**) Quantification of the percentage of PPARy-positive cells in treated tissues.

A specialized ex vivo organ culture (EVOC) model, the cResponse platform, was shown to predict drug response in breast cancer patients [26]. EVOC can preserve viable cancer tissues with the tumor’s intact microenvironment for 5–7 days. To test the possibility of inducing adipogenesis in heavily pretreated, metastatic triple-negative breast cancer patients, we compared PPARγ expression using IHC staining, following 5 days of ex vivo treatment. Pathological evaluation confirmed negative PPARγ staining in control sections and weak IHC staining in Pioglitazone- as well as in Cobimetinib-treated tissue sections. However, strong PPARγ expression was detected in the metastatic tumor section treated with a combination of Pioglitazone and Cobimetinib. Analysis of the percentage of cells positively stained for PPARγ indicated a >20 times increase in the percentage of positive cells (Figure 6). These results indicate a possible response of heavily pretreated metastatic TNBC to trans-differentiation treatment.

## 4. Discussion

Based on our previously published results, we aimed to study the class effect of FDA-approved drugs from the TZD family and MEK inhibitors on cancer cell-induced trans-differentiation into adipocytes. Utilizing established protocols and adipogenesis analysis, we demonstrate a broad class effect, thus suggesting the possible exchange between FDA-approved MEK inhibitors and TZDs according to toxicity profile and patient tolerability in future clinical trials. 

Interestingly, our results suggest that the described inhibitory effect of TGFβ on adipogenesis correlates with PPARγ downregulation but does not regulate C/EBPα expression. Further investigation is required to decipher the mechanism of TGFβ-mediated inhibition of adipogenesis in cancer cells. 

The aim of the current study was to evaluate the trans-differentiation class effect of MEK inhibitors and TZD. Our results demonstrate similar adipogenesis induction across tested drugs, leading to cancer cells’ conversion into post-mitotic terminally differentiated adipocytes, and thus supporting the class effect hypothesis. Intriguingly, Cobimetinib exhibited enhanced adipogenesis when compared to other MEK inhibitors, as observed by the earlier maturation of typical adipocytic lipid droplets. Future studies will investigate the mechanism and structure-function relationship of Cobimetinib compared to other MEK inhibitors in inducing adipogenesis in cancer.

We further demonstrated the utility of the cResponse platform for evaluation of initial adipogenesis induction as detected with PPARγ upregulation. While the combination treatment resulted in strong PPARγ staining, treatments with Cobimentinib or Pioglitazone alone were sufficient to slightly increase the expression of PPARγ. Our results reflect the high plasticity and trans-differentiation potential of the tumor tissue. The cResponse platform enables relatively long treatments for up to 7 days. Yet, to allow terminal differentiation of cancer cells into adipocytes, prolonged drug exposure is required. By evaluating early adipogenesis transcription factors in treated tissue, trans-differentiation induction can be determined. Here, we demonstrate the utility of the ex vivo approach to test the trans-differentiation potential for precision medicine. Thus, the cResponse platform can be utilized to assess the differentiation potential of various breast cancer subtypes. Additionally, it has the potential to be expanded to other cancer types, thereby enhancing personalized medicine approaches.

## 5. Conclusions

Our study confirms the class effect of PPARγ agonists and MEK inhibitors in promoting adipogenesis in breast cancer cells, highlighting their potential interchangeability in clinical applications and offering a promising option for patients with treatment-resistant TNBC. Given the role of cellular plasticity in various cancers, this approach could serve as a tumor-agnostic therapeutic strategy, potentially effective across multiple malignancies. Future clinical studies should focus on validating this approach, refining treatment protocols, and assessing its safety and effectiveness, while also exploring how it can be combined with standard treatments to overcome resistance in aggressive cancers.

## Data Availability

The original contributions presented in the study are included in the article/Supplementary Material; further inquiries can be directed to the corresponding authors.

## Supplementary Materials

The following supporting information can be downloaded at https://www.mdpi.com/article/10.3390/cells13171506/s1, Figure S1: The class effect contribution of MEK inhibitors to cancer adipogenesis in murine breast cancer cells is dose-dependent; Figure S2: MEK inhibitors decrease cell proliferation in TGFβ-treated murine breast cancer cells during trans-differentiation; Figure S3: Combination of Cobimetinib with Pioglitazone or Rosiglitazone overcomes the inhibitory effect of TGFβ on cancer adipogenesis.
