# Supplementary material for: Class Effect Unveiled: PPARγ Agonists and MEK Inhibitors in Cancer Cell Differentiation
Source: Cells. 2024 Sep 9;13(17):1506. doi: 10.3390/cells13171506 (PMC11394433; doi:10.3390/cells13171506)
Supplement: Supplementary file 1 [file cells-13-01506-s001.zip › cells-3151723-supplementary.pdf]

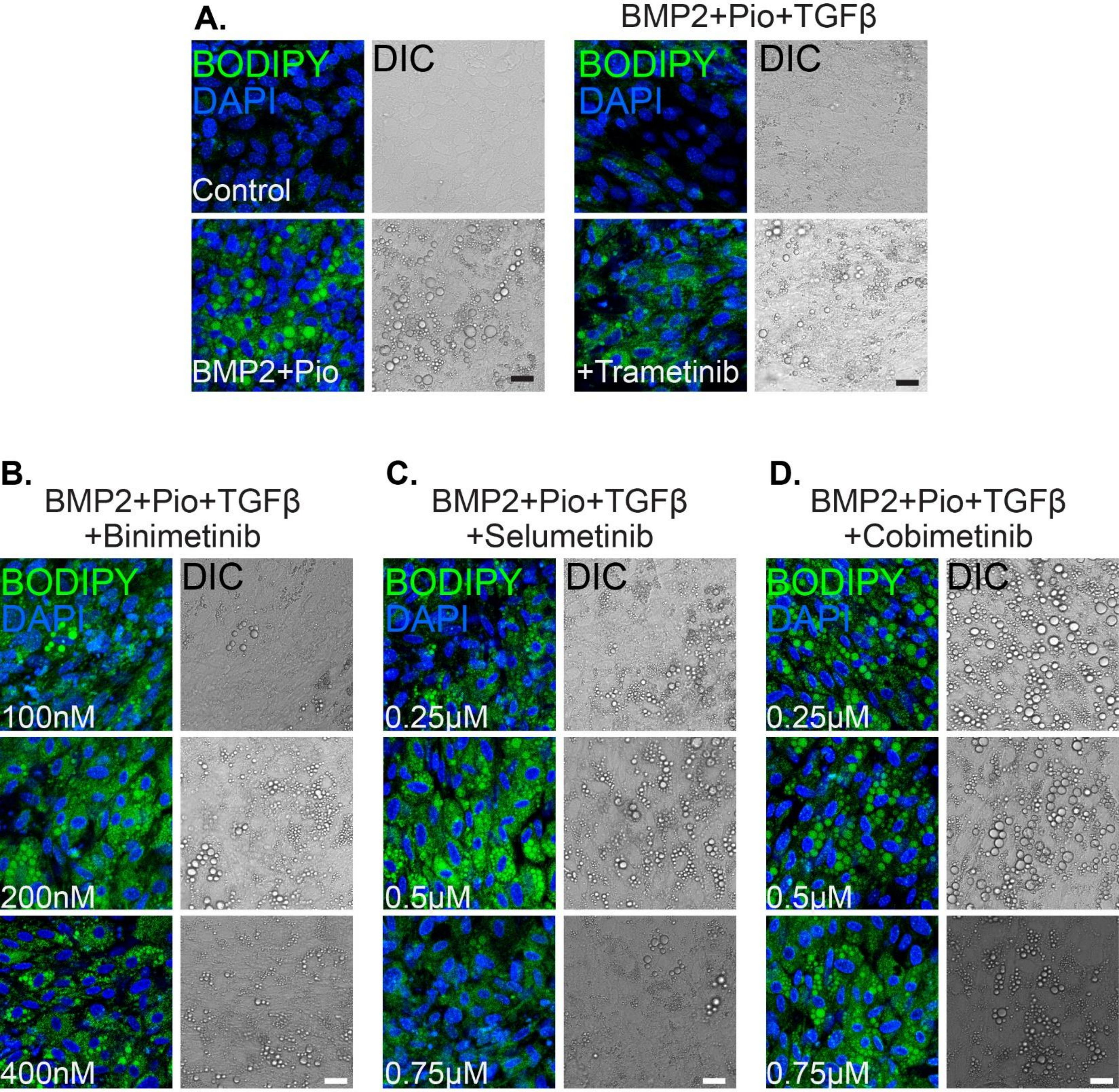

Figure S1: The class effect contribution of MEK inhibitors to cancer adipogenesis in murine breast cancer cells is dose-dependent. (A-D) MTΔECad cells were treated for 10 days for adipogenesis in the presence of TGFβ and different concentrations of MEK inhibitors (MEKi) as indicated. Control cells were treated with DMSO. Cells were stained with BODIPY to mark lipid droplets (green) and with DAPI (blue) and visualized with confocal microscope. DIC images of the same fields are shown on the right. Bars = 20μm.

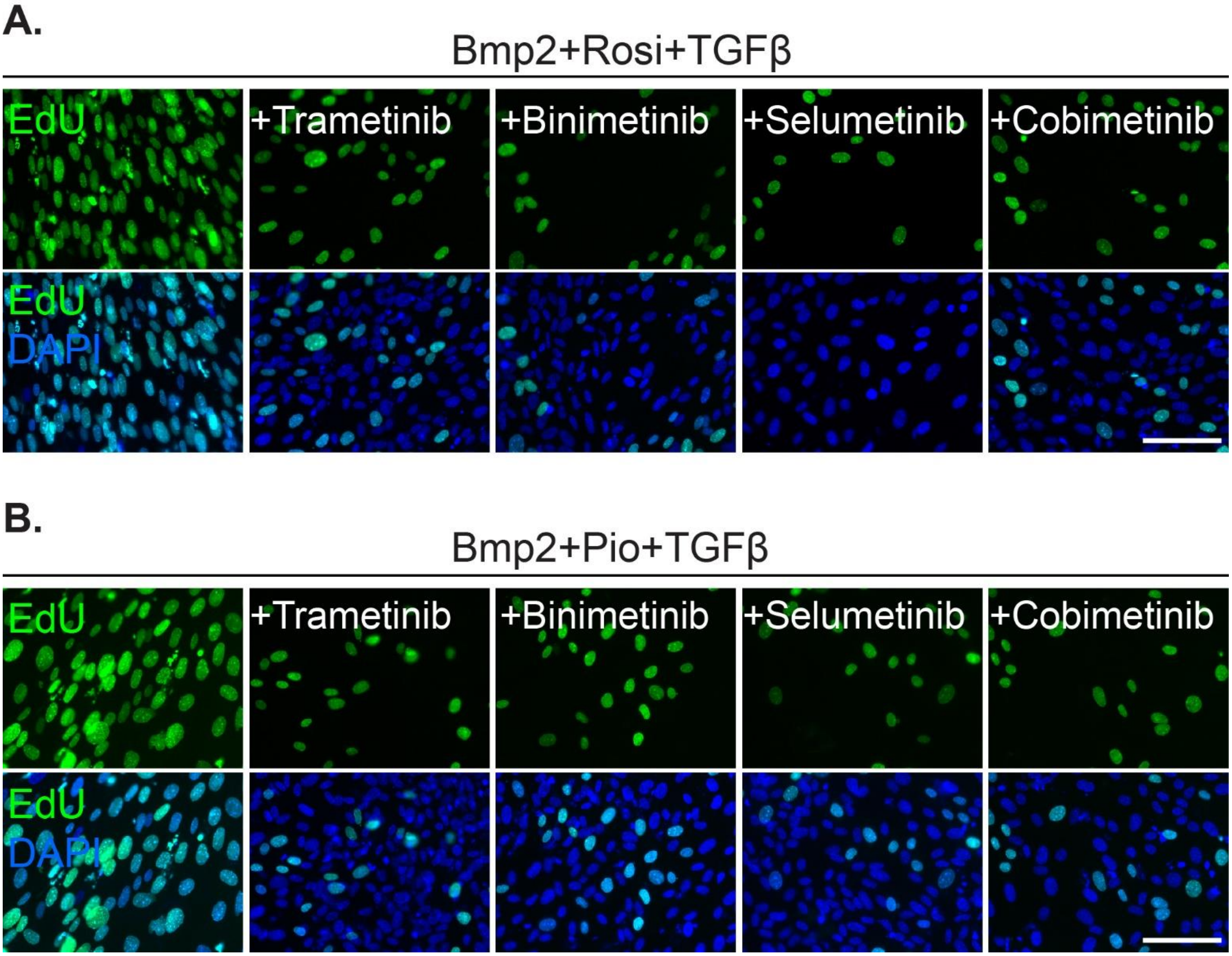

Figure S2: MEK inhibitors decrease cell proliferation in TGFβ-treated murine breast cancer cells during trans differentiation. Representative images of MTΔECad cells treated for 10 days with TGFβ, BMP2 and Rosiglitazone (Rosi) (A), or BMP2 and Pioglitazone (Pio) (B), with or without MEK inhibitors as indicated. At day 7 of adipogenesis treatment, cells were incubated with 5-ethynyl-20-deoxyuridine (EdU) for 72 hours to label proliferating cells (green). DAPI stain is shown in blue. Bars=100µm.

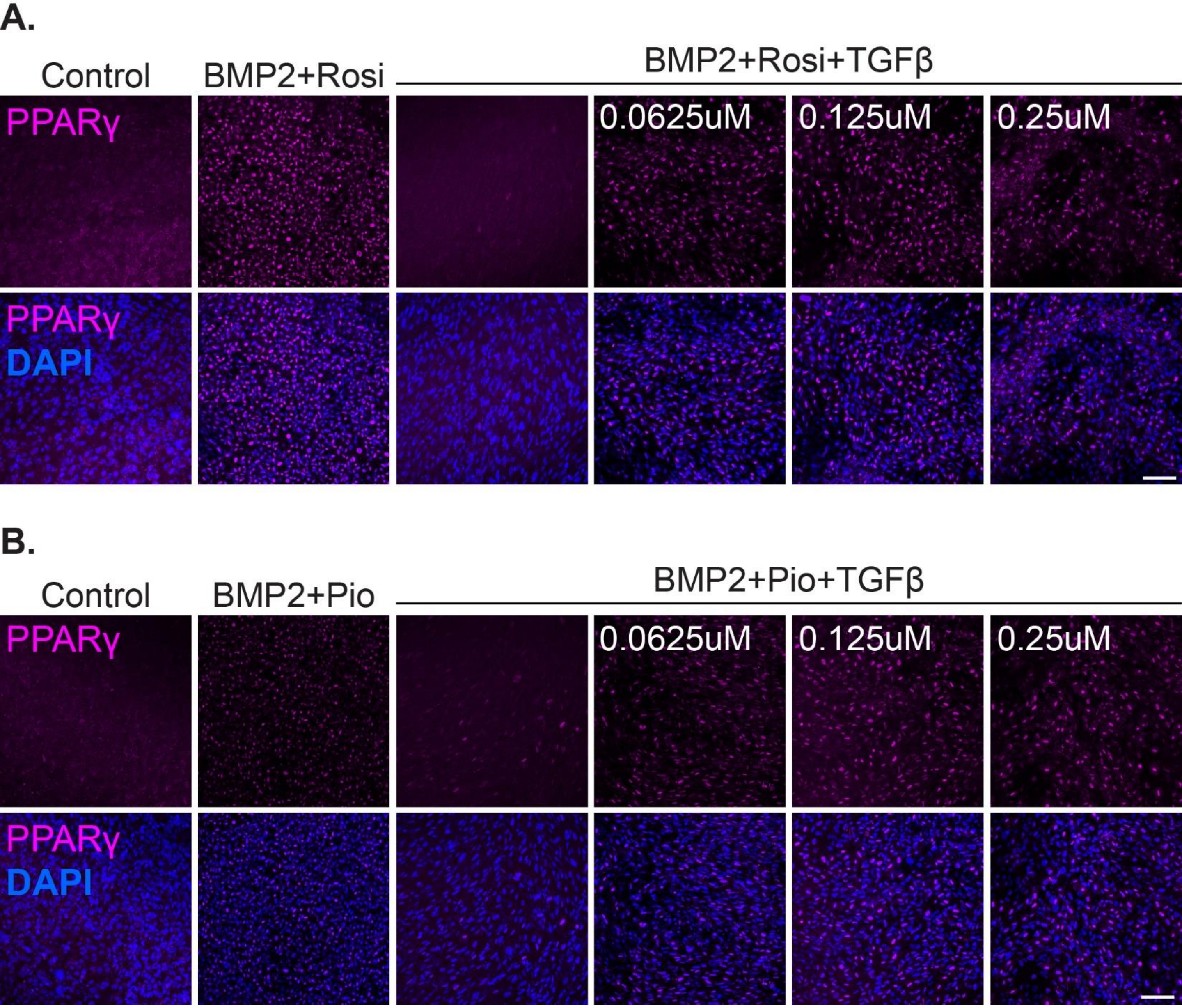

Figure S3: Combination of Cobimetinib with Pioglitazone or Rosiglitazone overcomes the inhibitory effect of TGFβ on cancer adipogenesis. MTΔECad cells were treated for 10 days with BMP2, Rosiglitazone (Rosi), (A) or Pioglitazone (Pio), (B), TGFβ and Cobimetinib in different concentrations as indicated. Control cells were treated with DMSO. Cells were immunostained with antibody against PPARγ (Magenta) and counterstained with DAPI (blue). Bars=100μm.
